# The State Board of Health of Illinois—An Example Worthy of Imitation

**Published:** 1883-03

**Authors:** 


### THE STATE BOARD OF HEALTH OF ILLINOIS—AN EXAMPLE WORTHY OF IMITATION.

In the last number of the REGISTER we commented favorably upon the showing made by the Illinois State Board of Health in its Fourth Annual Report, and then expressed the determination of considering some of the features of this report at greater length in the present issue; more especially those portions which relate to the functions of the board as the executive of the medical practice act of that State.

The subject of State regulation of the practice of medicine is extremely perplexing, but one which deeply concerns the medical profession of the whole country. In fifteen States of the Union some effort has been made, with varying degrees of success, to accomplish what seems to have been so skilfully wrought out in Illinois, and it is to the salient points in these laws and their practical working that we desire to invite special attention.

In 1877 a bill was enacted establishing a State board of health to consist of seven persons appointed by the governor with the advice and consent of the senate, to serve without compensation, excepting the secretary, who should receive a salary to be fixed by the board and all necessary traveling expenses incurred in the discharge of his duties. The duties of the board are prescribed in the act and, with one notable exception to be immediately stated, are similar to those which usually devolve upon such bodies. Practitioners are required to register somewhat after the manner observed in this State, and five thousand dollars per annum is appropriated to defray the expenses incident to the operations of the board.

At the same time an act to regulate the practice of medicine was passed and the board was charged with its execution. Under this act it is made the duty of the board to revise the diplomas of all medical practitioners within the State, for the purpose of determining whether or not they are genuine and if they have been lawfully conferred, and also to examine applicants for license to practice medicine within the State. The fee for the examination of a diploma, if genuine, is one dollar; if fraudulent, twenty dollars shall be collected from the person presenting it. The fee for the examination of non-graduates is five dollars to be returned in cases where the license is not granted. The board may refuse certificates to individuals guilty of unprofessional or dishonorable conduct, and it also has the power to revoke certificates for like cause. Itinerant vendors of medicines or medical appliances of any sort, are required by the act to pay to the board a license fee of one hundred dollars per month.

It will be observed that the distinguishing and a very important feature of this act is the provision making the State board of health the judge of the requisite qualifications for practicing medicine within the State, and it is likewise charged with the very responsible power of revoking licenses or refusing permission to practice for unprofessional or dishonorable conduct, and the board is made the sole judge of what constitutes unprofessional or dishonorable conduct. It is also empowered to decide upon the standing of medical colleges the diplomas of which may be presented for examination.

From the directory of institutions, prepared by the board, which are authorized to confer medical diplomas and licenses in the United States and Canada, we learn that there have been 175 organized since 1765. Of which number 110, are still in existence. The diplomas of 83 of these institutions have been presented to the board since its organization—68 of which were recognized as in good standing, and 15 were rejected absolutely or conditionally. There still remains 45 institutions—25 existing and 20 extinct—concerning the standing of which the board has not yet been called upon to decide.

Of this number of medical deploma-granting institutions Georgia is credited with nine; none of which are represented in Illinois, excepting the Medical College of

Georgia, at Augusta, which contributes five practitioners to that State. The board, however, reports this college as "probably extinct, as no reply has been received to repeated requests for information."

The board, in this report, declares that under the operation of the medical practice act so many quacks have been driven from Illinois into other States that these latter have been still further aroused to the necessity for self-protection from this evil, and an increasing number are now moving to secure the necessary legislation.

We would rejoice, if it were only possible, to place a copy of this admirable report in the hands of every physician within this State, for we feel confident that its critical perusal would serve to arouse the profession of Georgia from the lethargy into which its seems to have fallen and would, we doubt not, cause it to arise as a mighty giant in the plenetude of its combined power to drive the despoiler from our borders. United and determined the force which it could muster would be invincible; its requests would be granted, its demands would be conceded. But, alas! it is sleeping over its opportunities, and is suffering the stranger and the adventurer to rob it of its precious heritage. It is oblivious to its possibilities and the alien, unmolested, is bearing away its birth-right, while ignorance, incompetence and pretense run riot over these fair fields.

A homely proverb affirms "Where there is a will, there is a way." Let us realize the necessities of the hour. Let us have the will, and then the way will surely be found.

